# 126 novel mutations in Italian patients with neurofibromatosis type 1

**DOI:** 10.1002/mgg3.161

**Published:** 2015-07-07

**Authors:** Donatella Bianchessi, Sara Morosini, Veronica Saletti, Maria Cristina Ibba, Federica Natacci, Silvia Esposito, Claudia Cesaretti, Daria Riva, Gaetano Finocchiaro, Marica Eoli

**Affiliations:** ^1^ Molecular Neuro‐oncologyIRCCS Foundation, “C. Besta” Neurological InstituteMilanItaly; ^2^ Developmental NeurologyIRCCS Foundation, “C. Besta” Neurological InstituteMilanItaly; ^3^ Medical GeneticsIRCSS Foundation, Cà Grande‐Ospedale Maggiore PoliclinicoMilanItaly

**Keywords:** Diagnostic criteria, mutation database, neurofibromatosis type 1 (NF1), novel mutations

## Abstract

Genetic analysis of Neurofibromatosis type 1 (NF1) may facilitate the identification of patients in early phases of the disease. Here, we present an overview of our diagnostic research spanning the last 11 years, with a focus on the description of 225 *NF1* mutations, 126 of which are novel, found in a series of 607 patients (513 unrelated) in Italy. Between 2003 and 2013, 443 unrelated patients were profiled by denaturing high pressure liquid chromatography (DHPLC) analysis of 60 amplicons derived from genomic *NF1* DNA and subsequent sequencing of heterozygotic PCR products. In addition, a subset of patients was studied by multiplex ligation‐dependent probe amplification (MLPA) to identify any duplications, large deletions or microdeletions present at the locus. Over the last year, 70 unrelated patients were investigated by MLPA and sequencing of 22 amplicons spanning the entire *NF1* cDNA. Mutations were found in 70% of the 293 patients studied by DHPLC, thereby fulfilling the NIH criterion for the clinical diagnosis of NF1 (detection rate: 70%); furthermore, 87% of the patients studied by RNA sequencing were genetically characterized. Mutations were also found in 36 of the 159 patients not fulfilling the NIH clinical criteria. We confirmed a higher incidence of intellectual disability in patients harboring microdeletion type 1 and observed a correlation between a mild phenotype and the small deletion c.2970_2972delAAT or the missense alteration in amino acid residue 1809 (p.Arg1809Cys). These data support the use of RNA‐based methods for genetic analysis and provide novel information for improving the management of symptoms in oligosymptomatic patients.

## Introduction

Neurofibromatosis type 1 (NF1) is a human autosomal dominant disorder that affects approximately 1 in 3500 individuals worldwide (Carey et al. 1986; Friedman 1999). The most common features of NF1 are pigmentary abnormalities such as café‐au‐lait macules (CALs) and skinfold freckling, Lisch nodules (LNs), cutaneous and plexiform neurofibromas (PNs), optic gliomas, and bone lesions. These symptoms are age‐dependent (with a full clinical manifestation typically by 12 years of age) and present high variability in expressivity, even among affected members of the same family (Jett and Friedman 2010).

NF1 is caused by mutations in the neurofibromin gene (17q11.2.5‐7; NM_000267.3), which encodes a negative regulator of the Ras guanosine triphosphate (GTP)ase proteins and acts as a tumor suppressor gene. The detection of mutations in the NF1 gene is complex due to the large size of the gene (>350 kb), the presence of pseudogenes, the lack of hot spots, and a high mutation rate (50% of cases are sporadic and stem from de novo mutations; Cawthon et al. 1990; Huson 2008).

To date, small changes are most frequently identified in *NF1* (approximately 80–90%) and often cause premature stop codons. Between 5% and 10% of these changes were characterized by microdeletion of the entire NF1 gene and contiguous genes (1–1.4 Mb) (Kluwe et al. 2004). Insertion and copy number alterations were reported less frequently in the literature (Kehrer‐sawatzki et al. 2014). This wide mutational spectrum and the complex molecular features of the NF1 gene have been a source of difficulty in the identification of genotype–phenotype correlations.

At present, the diagnosis of NF1 is defined by clinical criteria described by the NIH in 1988 (Conference, 1988). However, children under 12 years without affected family members often share clinical features with “Rasopathy” syndromes such as Legius (Brems and Legius 2013; Rauen 2013). Thus, early clinical and molecular diagnosis of NF1 is challenging and requires improvement.

Between 2003 and 2013, we performed an *NF1* mutation analysis using a DNA‐based approach: multiplex ligation‐dependent probe amplification (MLPA) analysis was used to identify deletions and insertions in the NF1 gene, followed by denaturing high pressure liquid chromatography (DHPLC) and DNA sequencing to identify small pathogenic changes. We recently updated our protocol to a more sensitive approach utilizing both DNA and RNA to decrease the time of the analysis and increase mutation detection. Furthermore, RNA sequencing may help detect mutations affecting splicing that are not located at canonically conserved GT/AG splice sites (Messiaen et al. 2000; Wimmer et al. 2007; Valero et al. 2011).

Here, we report the mutational spectrum of 607 Italian patients referred to our center for the molecular analysis of NF1 over the last eleven years (2003–2014). Notably, we report 126 new alterations identified in NF1 patients displaying a partially or fully developed phenotype, which could help clinicians with patient management and genetic counseling.

## Materials and Methods

### Patient population

Patients were referred to our Institute from twenty northern Italian centers. All gave written informed consent prior to genetic analysis.

A totals of 537 people were screened in our Institute between May 2003 and August 2013 for *NF1* mutations by a DNA‐based approach including MLPA, DHPLC analysis and DNA sequencing; 443 of the patients were unrelated, whereas 94 were related. The median age was 22, and females and males spanned the same range. 293 of the unrelated patients fulfilled NIH criteria. The median age was 22 years (range: 6 months to 67 years); 42% were less than 12 years of age (122/293), and 50% were less than 18 years of age (146/293).

The 94 individuals with relatives included in the study were representative of 57 Italian families. Thirty‐four of these 57 families were investigated for affected members, and 23 of the 57 families were used to verify the pathogenicity and de novo origin of the detected alteration.

Seventy unrelated patients were screened for *NF1* mutations by RNA‐sequencing after prescreening by MLPA between September 2013 and August 2014. The median age was 27 years (range: 4 months to 70 years), and contained 64% females; 87% of the patients (61/70) manifested the clinical features of NF1.

### DNA extraction, RNA extraction and retro‐transcription

DNA was isolated from ethylenediamine tetraacetic acid (EDTA) blood samples using a Gentra^®^ Puregene^®^ Blood Core Kit B (Qiagen, Venlo, Netherlands Carlsband, California, USA). RNA samples were collected in Tempus Blood RNA Tubes (Life Technologies, Carlsbad, CA) and extracted with a Tempus^™^ Spin RNA Isolation Kit within 4 days. DNase treatment with Absolute RNA Wash Solution was performed for all samples during the RNA extraction protocol. RNA samples (1 *μ*g) were reverse‐transcribed using 50 units of High‐Capacity cDNA Reverse Transcription mix (Life Technologies) and 20 units of RNAse inhibitor (Ambion^®^, Austin, TX). Beta‐2‐Microglobulin amplification was used as a quality control for retro‐transcription.

### MLPA analysis

Patients' DNA was analyzed by MLPA with NF1 MLPA SALSA P081 and P082 (MRC Holland, Amsterdam, The Netherlands). MLPA was performed routinely after July 2008, and a retrospective analysis was conducted for negative patients. P081/P082‐positive patients with a deletion of the entire NF1 gene were screened again with the MLPA P122 SALSA kit. The results obtained with a ABI Prism 3130 Genetic Analyzer (Life Technologies) were analyzed using Coffaliser.Net Software (MRC Holland).

### Microarray‐based comparative genomic hybridization (aCGH)

Abnormalities identified by MLPA were subsequently tested by CGH Agilent array (protocol CGH v.6.0). A custom‐designed 8 × 60 K array (Agilent Technologies, Santa Clara, CA) was used to detect exon deletions and duplications in the NF1 gene (data available on request). CGH array experiments were performed by the department of Human Pathology at Pavia University.

### DHPLC analysis

Genomic DNA from patients testing negative by MLPA was analyzed by DHPLC prior to December 2013. The NF1 gene was amplified by PCR in 60 amplicons of approximately 400 bp each. PCR products were analyzed on a DHPLC Wave Transgenomic 3500HT (Transgenomic^®^, Crewe, UK) equipped with a DNASep column (Transgenomic^®^). The oven temperatures for optimal heteroduplex separation were determined using WAVEmaker software v4.1.40 (Transgenomic^®^). Primers and annealing temperature were improved from Han et al. 1999 and Upadhyaya et al. 2004 (data available on request). PCR products displaying a heterozygous pattern were sequenced using an ABI Prism 3130 Genetic Analyzer (Life Technologies) to identify mutations.

### DNA sequencing

Sequencing analysis was performed on positive amplicons obtained via DHPLC. PCR reactions were performed using Taq Gold Polymerase^®^ (Applied Biosystems, Foster City, CA). The sizes of PCR products were verified by electrophoresis on a 2% agarose gel. PCR products were purified using ExoSAP‐IT^®^ (USB Corporation, Cleveland, OH) according to the manufacturer's protocol and were sequenced in both directions using an ABI PRISM BigDye terminator sequencing kit v1.1 (Life Technologies) on an ABI Prism 3130 Genetic Analyzer (Life Technologies). Primers used for amplification and sequencing were the same as those used for DHPLC analysis. Bidirectional DNA sequences were compared to normal DNA and to NM_000267.3 reference sequence (NC_000017.10). *NF1* exon numbering followed that used in the NCBI Reference Sequence (NG_009018.1) and included exons 1 to 58 (skipping exon 31, formerly referred to as exon 23a). Family members were usually studied by direct sequencing for the known mutation only.

### cDNA amplification and sequencing

*NF1* cDNA was fully amplified in 22 overlapping fragments ranging from 400 to 560 bp (Valero et al. 2011). Primer sets were designed according to Valero et al. with the exception of segment F16 (exon 37 to 39). cDNA variations were confirmed at the DNA level, and exons 1 and 23a were typically sequenced, as described above, at the DNA level to test for GC‐rich regions.

### Prediction of impacts at the protein and mRNA levels

The possible effects on NF1 gene and protein of the genetic variations identified only in DNA were predicted based on different databases and prediction sites including Mutation Taster (http://www.mutationtaster.org) by simultaneously querying different databases, such as the NCBI SNP database (dbSNP), the 1000 Genomes Project (TGP), disease variants from dbSNP (ClinVar) and the Human Genome Mutation Database (HGMD) (Schwarz et al. 2010). The HGMD (http://www.hgmd.cf.ac.uk/ac/index.php ‐ Institute of Medical Genetics, Cardiff, Wales, UK) and LOVD (Leiden Open Variation Database‐ http://www.LOVD.nl/NF1) (Fokkema et al. 2011; van Minkelen et al. 2014) databases were usually interrogated to verify whether the mutation was novel. PolyPhen‐2, (http://genetics.bwh.harvard.edu/pph2/) (Adzhubei et al. 2010) was used to predict the pathogenicity of novel missense alterations by evaluating the functional and structural impacts of an amino acid substitution. The possible effects on mRNA were evaluated with tools including splice site prediction by neural network (www.fruitfly.org/seq_tools/splice.html) (Reese et al. 1997), the Human Splicing Finder (HSF; http:www.umd.be/HSF/) (Desmet et al. 2009) and exonic splicing enhancer (ESE) Finder (http://rulai.cshl.edu/cgibin/tools/ESE3/esefinder.cgi?process=home) (Cartegni et al. 2003; Smith et al. 2006).

### Clinical data and data analysis

Clinical data for patients were obtained through a clinical questionnaire completed by the referring physician. The chi‐squared test or Fisher's exact test was used to test for differences in mutation distribution among the four protein domains of neurofibromin.

Genotype–phenotype correlations were studied using multiple logistic regression models, with covariates of gender and age at the time of genetic testing. Odds ratios (OR) and 95% confidence intervals were calculated when there was a significant effect. The Bonferroni method was used to correct for multiple testing (significance level *P* = 0.003). Clinical features were considered individually for each type of mutation. Statistical analyses were performed with the software program SPSS 22.0 for IBM (SPSS Inc., Chicago, IL).

## Results

### NF1 cases detected by a DNA approach (2003–2013)

**Figure 1:**
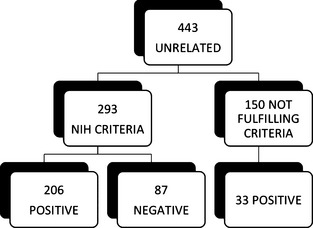
Summary of unrelated patients studied by DNA approach (2003–2013).

During the first 10 years of our diagnostic study, 443 unrelated patients were referred to the C. Besta Neurological Institute for clinical genetic testing of the NF1 gene (Fig. 1).

Among these 443 unrelated patients, only 293 fulfilled the NIH criteria for an NF1 diagnosis. The DNA‐based protocol permitted the detection of pathogenic mutation in 206 of the 293 patients, fulfilling the clinical criteria (detection rate 70%). MLPA analysis distinguished 20 of these 206 mutations (12 microdeletions, 6 intragenic deletions and 2 intragenic duplications), whereas DHPLC and DNA sequencing detected the other 186 mutations.

The DNA approach did not detect a pathogenic mutation in 87 of the 293 patients presenting with clinical features of NF1. Further characterization showed that one young patient had a *SPRED1* mutation (Legius syndrome; Brems et al. 2007). Three patients were characterized by benign variants: the c.8436T>C variant (p.Asn2812Asn; NF1_248), reported in LOVD as a silent variation with unknown pathogenicity, and the c.5172G>A variant (p.K1724K; NF1_92 and NF1_181), a silent variation described by Fahsold et al. 2000 at the DNA level and studied by Nementhova et al. at the RNA level (Fahsold et al. 2000; Nemethova et al. 2013). In two patients, we obtained clinical evidence of segmental NF1, probably due to mosaicism (NF1_40 and NF1_122). In addition, we reexamined 16 of the 83 negative cases with available RNA techniques. By RNA sequencing, we detected a pathogenic mutation in 12 of these 16 cases (75%). Overall, the pathogenic mutation in 71 patients went undetected despite the fulfillment of clinical criteria.

DNA analysis identified 33 mutations among 150 of the 443 that did not fulfill clinical criteria at the time of the study. Patients with incomplete clinical features included 98 of the 150 (65%) under 18 years of age (85 of the 98 under 12 years – 87%) and 52 adults (Table S1). We detected a pathogenic mutation in 6 of the 52 adults (11% – median age 39 years, range: 34–43 years) presenting with partial clinical features: two had neurofibromas only (NF_46 and NF1_552), 2 had fewer than 6 CALs or scoliosis and gliomas (NF1_212 and NF1_355, respectively), and 2 had learning disabilities (LD) and neurofibromas (NF1_19) or CALs (NF1_553) (Table 1).

**Table 1:** NF1 adults not fulfilling NIH clinical criteria

Patients ID	Age	Clinical features	Mutation	Reference
NF1_46	43	Neurofibromas	c.1466A>G	Osborn and Upadhyaya (1999), Messiaen et al. (1999)
NF1_552	39	Neurofibromas	c.7994A>G	Novel
NF1_212	34	CALs <6 and glioma	c.2291T>C	Fahsold et al. (2000)
NF1_355	42	Scoliosis and glioma	c.1062 + 113A>G	Novel
NF1_19	35	LD and neurofibromas	c.3916C>T	Park et al. (1998)
NF1_553	41	LD and CALs	c.7240 A>T	Novel

CALs, Café‐au‐lait; LD, learning disabilities.

Children under 12 years of age (26/33 – 79%) at the time of the study presented only with CALs in various numbers and with other minor clinical features (i.e., learning difficulties, Ubo's, hypertelorism, angiomas, or sclerosing cholangitis) (Table S1). Twelve children under 12 developed other major features such as lentigo, LNs and optic pathway gliomas.

94 individuals were related cases distributed among 57 Italian families. Of the 23 families screened for the presence of an alteration, a de novo mutation was found in 14 cases (60.8%).

### NF1 cases detected with MLPA and an RNA sequencing approach (2013–2014)

**Figure 2:**
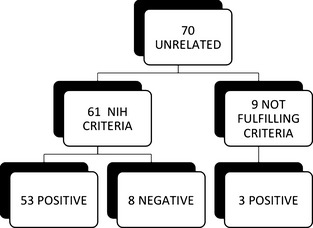
Summary of unrelated patients studied by RNA approach (2013–2014).

During the last year of the study, we updated our diagnostic methodology and took an RNA‐based approach to characterize 70 unrelated cases. This approach enabled us to identify 56 of the 70 positive cases: MLPA prescreening identified 5 variations (4 type 1 microdeletion and 4 intragenic deletions), and RNA sequencing detected another 49 positive cases (Fig. 2). In addition, 61 of the 70 patients fulfilled the NIH criteria. Thus, the detection rate was 87% (53/61). We could not detect any mutation in 8 clinically defined cases. Moreover, the RNA‐based protocol detected a pathogenic mutation in individuals NF1_556, NF1_658 and NF1_688, who did not fulfill NIH criteria possibly due to their young age (<2 years).

### Characterization of the mutations

**Figure 3:**
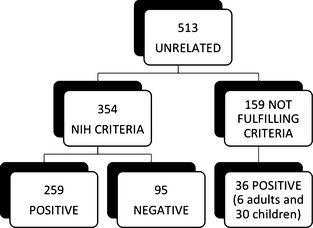
Summary of all unrelated patients studied (2003–2014).

**Figure 4:**
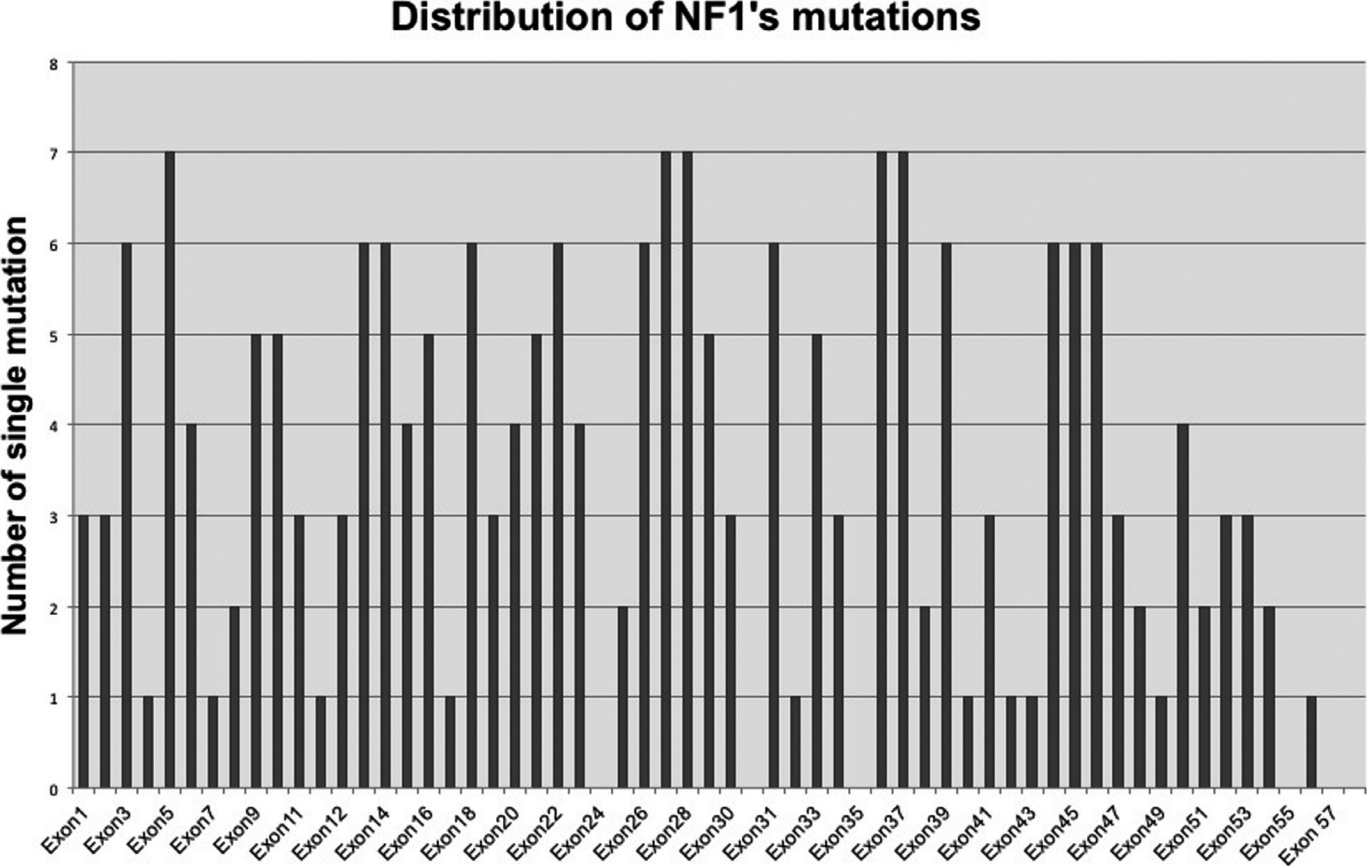
Distributions of the 206 single small mutations identified for each NF1 exon (17q11.2.5‐7; NM_000267.3).

Of the 443 unrelated patients investigated by DNA sequencing and the 70 unrelated patients analyzed within the last year (513 cases, overall), we identified 225 single mutations (Fig. 3). Nineteen mutations (8%) were gross deletions (17/19) or duplications (2/19) of one or more exons. The remaining 206 mutations (92%) were small changes spread among all *NF1* exons (Fig. 4). No hot spot region was identified. The exons most frequently hit by alterations (7 single mutations) were 5, 27, 28, 36 and 37. Twenty‐two amplicons contained between 4 and 6 alterations; 27 exons displayed 1 to 3 changes, whereas exons 24, 23a, 35, 43, 55, and 57 were unaffected (Fig. 4).

Overall, 87% of single mutations were unique (i.e., only observed in one unrelated patient or family; 196/225). The remaining 29 mutations (13%) were observed more than once. Exons 5, 13, 28, 37, and 45 were frequently mutated in our cohort. The most frequent mutation, identified in 9 unrelated patients, was c.1466A>G, p.Tyr489* (exon 13) (Table 2) (Messiaen et al. 1999; Osborn and Upadhyaya 1999). A type 1 microdeletion was found in 11 patients, and 2 unrelated patients presented microdeletion type III.

**Table 2:** Variable clinical features in patients with the same mutation

Patient ID	Age at diagnosis	Mutation	Clinical features	Fulfilling NIH criteria
NF1_46	43	c.1466A>G	3 NFs	No
NF1_48	1	c.1466A>G	CALs, AF	Yes
NF1_157	59	c.1466A>G	CALs, AF, NF, lymphoma	Yes
NF1_199	39	c.1466A>G	CALs, AF, NF, LN	Yes
NF1_229	51	c.1466A>G	CALs, NF, LN, GB	Yes
NF1_245	4	c.1466A>G	CALs, AF	Yes
NF1_279	11	c.1466A>G	CALs, AF, LN	Yes
NF1_712	12	c.1466A>G	CALs, LN	Yes
NF1_775	24	c.1466A>G	CALs, AF, NF	Yes

CALs, Café‐au‐lait; NF, neurofibromas; AF, axillary freckling; OG, Optic glioma; GB, Glioblastoma; LN, Lisch nodules.

In total, 126 of the 225 mutations (56%) were novel (i.e., not present in HGMD and LOVD databases). All novel mutations identified herein have been deposited in the LOVD database (http://www.LOVD.nl/NF1) and described according to HGSV recommendations. All sites of missense mutations were sequenced in 100 healthy individuals to confirm that the mutation was not a single nucleotide polymorphism (SNP). The 126 novel mutations are summarized in Table S2.

**Figure 5:**
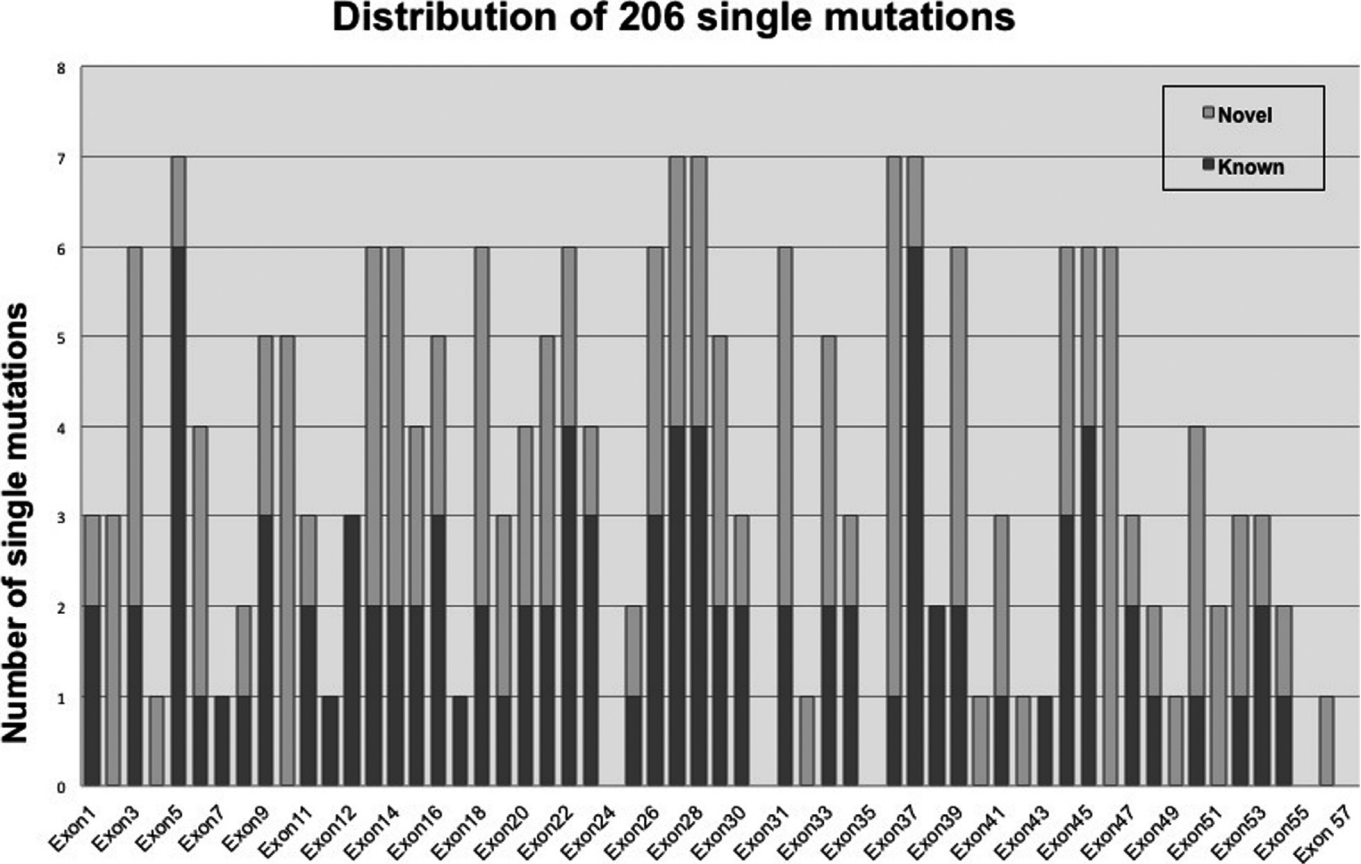
Distributions of the 206 single small mutations divided in Known (95) and Novel mutations (110) for each NF1 exon. Gray bars represent the novel mutation while the black bar the known alterations (17q11.2.5‐7; NM_000267.3).

Of the 126 novel mutations, 110 were small changes and 16 were aberrant deletions/duplications of one or more exons (Table 4). The 110 small mutations were distributed along all *NF1* exons, from 1 to 58 (Fig. 5).

Four NF1 protein domains have been identified: a Cystein and Serine Rich Domain (CSRD; amino acids 543–909), a Gap Region Domain (GRD; amino acids 1168–1530), a Leucine Zip Domain (LZD; amino acids 1543–1550), and a C‐terminal Domain (CTD; amino acids 2260–2818) containing the Nuclear Localization Site (NLS; amino acids 2534–2550) and Tyrosine Kinase Recognition sites (TRS; amino acids 2549–2556). We observed a trend in the localization of the novel mutations to the GRD (18/110 – 16%), CTD (20/110 – 18%) and CSRD (14/110 – 13%). No novel mutations affected the LZR or the TRS site, although one was located in the NLS domain (exon 51). The remaining 53% of mutations fell within exons not yet linked to a biological function (Table S3).

**Figure 6:**
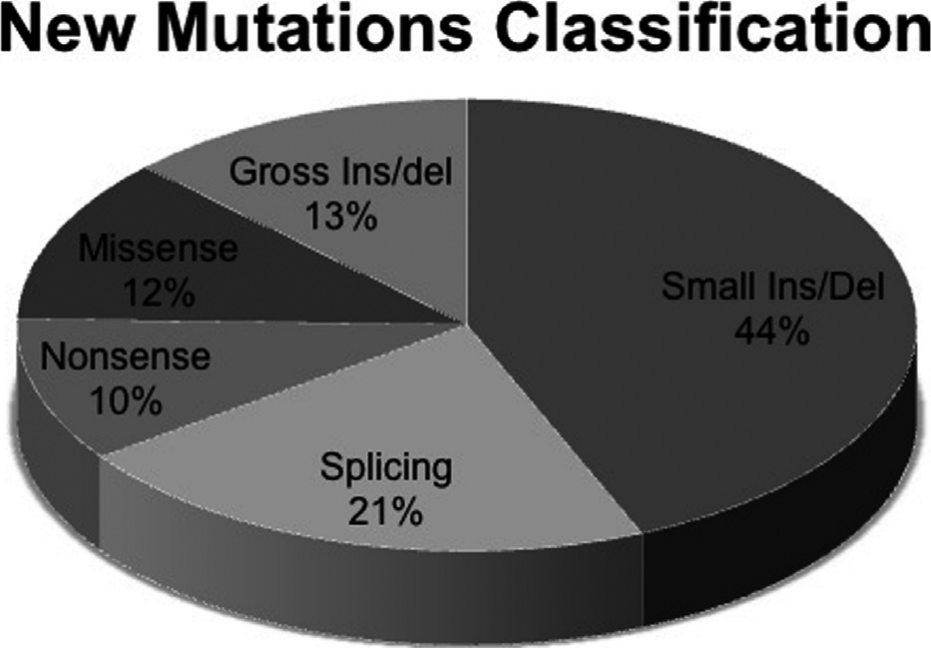
The classification of 126 novel NF1 mutations (17q11.2.5‐7; NM_000267.3).

The majority of the identified mutations consisted of small changes (110/126 – 87%), such as small deletions and insertions (44% – 55/126) causing a frameshift or a premature stop codon. Others were splicing (21% – 27/126), missense (12% – 15/126) and nonsense (10% – 13/126) mutations (Fig. 6). Atypical microdeletions and deletions/insertions of one or more exons represented 13% of mutations (16/126; 4% atypical microdeletion and 9% deletion/duplication).

Deletions of 1 or 2 nucleotides were the most frequent alteration (26/55). However, patient NF1_459 lost 7 nucleotides in the exon 15, and NF1_156F lost 15 nucleotides of exon 36, whereas only 2 patients had in‐frame deletions (NF1_82; NF1_156F). Fourteen of 55 patients harbored an insertion or duplication of 1 or 2 nucleotides. We identified the duplication of part of exon 44 in one patient (NF1_6). In 4 cases, deletions and insertions of new nucleotides caused a frameshift (NF1_384, 339, 614), and in one case an in‐frame alteration (NF1_314). The other 6 patients had deletions or duplications of more than 3 nucleotides (see Table S2 for more details).

#### Single‐nucleotide change mutations: nonsense, missense and silent mutations

Of the novel mutations identified, 13 (10%) created stop codons and produced a truncated protein, whereas 15 were classified as missense. We used PolyPhen software to predict whether the amino acid variations were pathogenic by examining their conservation during evolution. Four of 13 missense mutations found by DNA analysis are predicted to cause splicing errors: NF1_208 and NF1_197 hit the splice site consensus sequence, whereas NF1_434/640 and NF1_322 fell within ESE sequences that influence correct splicing.

#### Splicing mutations: effect on mRNA

The splicing mutations identified herein predominantly affected consensus splice sites (21/27 – 78%); 17 affect the donor site (5′SS) and 4 the acceptor site (3′SS).

NF1_355 has a deep intronic alteration in intron 9 (c. 1062 + 113 A>G). In silico analysis (Fruitfly, see M&M) showed that this mutation creates a new donor site. This results in the inclusion of a cryptic exon in the mRNA transcript and the formation of a truncated protein of 366 aa. Meanwhile, NF1_556 (c.3748C>A) created a new acceptor site inside exon 28, and NF1_416 (c.2410‐18 C>G) resulted in the formation of a new acceptor site 18 nucleotides upstream of the canonical site. These mutations were confirmed by RNA analysis.

#### Microdeletion and intragenic deletion/duplication of one or more exon

Sixteen patients had deletions or exon duplications that were revealed by MLPA (Table 3). Gross rearrangements were confirmed by CGH‐array analysis. We found 5 of the 16 atypical microdeletions that involved the NF1 gene and the flanking centromeric and telomeric regions of chromosome 17q11.2. These deletions ranged from 0.8 to 1.8 Mb in size and included different genes such as NUFIP2, BLMH, CRLF3, and RFN135 at the centromeric region, and RAB11TIP4, UTP6, SUZ12, MYOD at telomeric region. Notably, 2 atypical deletions were familial cases (NF1_31 and NF1_582) (Table 3A). Moreover, we detected new intragenic deletions (9/16) and duplications (2/16) (Table 3B and C). Intragenic deletions spanned over 35 kb and included exon 1. Two distinct familial cases presented the same internal deletion of exon 34, which was transmitted to offspring: NF1_373 to his daughter NF1_374, and NF1_95 to his son NF1_96. The other 7 cases reported deletions affecting multiple exons. Two intragenic duplications were also found: NF1_602 (exons 28/29; 0.3 Kb) and NF1_295 (exons 13/31; 45 Kb).

**Table 3:** (A) New atypical microdeletion; (B) deletions and (C) duplication of one or more exons

Patient	S/F	Deleted	Type of deletions	Array‐CGH hg18 assembly deletion 17q11.2
(A) New atypical microdeletion				
NF1_31^a^	F	NUFIP2 ‐ ex1 NF1	Atypic (1.8 Mb)	24,634,917–26,461,947
NF1_226^a^	S	CRLF3‐NF1‐RAB11FIP4	Atypic (0.871 Mb)	26,173,551–27,044,653
NF1_505	S	BLMH‐NF1	Atypic (1.1 Mb)	
NF1_582	F	RNF135‐NF1	Atypic (0.4 Mb)	
NF1_724	S	Exon6 NF1‐ MYOD	Atypic (>1.6 Mb)	

Patient	S/F	Deleted	Type of deletions	Array‐CGH hg18 assembly deletion 17q11.2
(B) Deletions of one or more exons				
NF1_(95^a^‐96)	F	34	Single exon (1.215 kb)	26,612,509–26,613,724
NF1_(373‐374)	F			
NF1_267^a^	S	14–31	Multiple exons (35 kb)	29,545,984–29,580,921
NF1_(451‐494)	F	13–19	Multiple exons (13 kb)	
NF1_377	S	30/31	Multiple exons (4 kb)	
NF1_468	F	1 + promoter	Single exon (4 kb)	
NF1_521	NA	6–10	Multiple exons (19.6 kb)	
NF1_650	S	10–34	Multiple exons (60.6 kb)	
NF1_670	S	10‐57	Multiple exons (173 kb)	
NF1_785	S	30‐35	Multiple exon (16.3 kb)	

Patient	S/F	Duplication	Type of duplication	Array‐CGH‐hg18 assembly duplication 17q11.2
(C) Duplication of one or more exons				
NF1_295^a^	S	13/31	Multiple exon (45 kb)	29,536,361–29,581,490
NF1_602	F	28/29	Multiple exon (0,3 kb)	

These deletions were sequenced and none of the corresponding exons were found to carry a point mutation within the corresponding MLPA probe. NA, not available; F, familiar; S, sporadic.

a Deletions confirmed by CGH‐array.

### Clinical data

We reviewed the clinical data of 207 patients with *NF1* mutations (shown in Table 4).

**Table 4:** Mutated patients clinical data

Patient	Total	Under 12 year	Over 12 year
Number	207	63	144
Café‐au‐lait	193 (93.2)	63 (100)	130 (90.3)
Freckles	149 (72)	53 (84.1)	96 (66.7)
Lisch nodules	100 (48.3)	15 (23.8)	85 (59)
Neurofibromas	135 (65.2)	14 (22.2)	121 (84)
Plexiform neurofibromas	35 (16.9)	9 (14.3)	26 (18.1)
Optic glioma	34 (16.4)	9 (14.3)	25 (17.4)
Osseous lesion	10 (4.8)	3 (4.8)	7 (4.9)
NF1 familiar	64 (30.9)	11 (17.5)	53 (36.8)
No minor feature	73 (35.3)	20 (31.7)	53 (36.8)
Tumors	34 (16.4)	9 (14.3)	25 (17.4)
Scoliosis	31 (15)	4 (6.3)	27 (18.8)
Macroencephaly	60 (29)	26 (41.3)	34 (23.6)
Short stature	14 (6.8)	6 (9.5)	8 (5.6)
Learning disability	26 (12.6)	8 (12.7)	18 (12.5)
Special education	3 (1.4)	0 (0)	3 (2.1)
Mental retardation	18 (8.7)	7 (11.1)	11 (7.6)
Speaking problems	7 (3.4)	5 (7.9)	2 (1.4)
Behavior problems	5 (2.4)	2 (3.2)	3 (2.1)
Epilepsy	9 (4.3)	3 (4.8)	6 (4.2)
Other	40 (19.3)	18 (28.6)	22 (15.3)

Parentheses values are expressed in percent.

The percentage of major clinical phenotypes is in accordance to previous reports (Tonsgard 2006; Jett and Friedman 2010). When patients under 12 years of age were excluded, the incidence of neurofibromas and LNs decreased (Huson 2008). The majority of patients reviewed fulfill the NIH criteria (*n* = 171), whereas 30 did not show two of the six major features of NF1. Minor NF1 features were observed in both adults and children (Table 4).

No new genotype–phenotype correlations were observed (Table 5). Patients with microdeletions presented a higher frequency of mental retardation than patients with other mutations after the Bonferroni correction reached statistical significance (*P* = 0.002 OR 7.95, 95% CI: 2.03–31.01). Moreover, missense mutations were associated with minor neurofibromas (multiple and plexiform) more often than truncating mutations, although statistical significance was not reached. No association with gender and/or age was observed in the regression analysis.

**Table 5:** Genotype–phenotype correlations

Patient	Intragenic mutation	Microdeletion type 1	*P*‐value
Number	179	11	
Café‐au‐lait	167 (93.3)	11 (100)	NS
Freckles	128 (71.5)	8 (72.7)	NS
Lisch nodules	84 (46.9)	5 (45.5)	NS
Neurofibromas	119 (66.5)	5 (45.5)	NS
Plexiform neurofibromas	29 (16.2)	3 (27.3)	NS
Optic glioma	34 (19)	0 (0)	NA
Osseous lesion	8 (4.5)	0 (0)	NA
NF1 Familiar	56 (31.3)	0 (0)	NA
No minor feature	66 (36.9)	1 (9.1)	NS
Tumors	28 (15.6)	2 (18.2)	NS
Scoliosis	28 (15.6)	1 (9.1)	NS
Macroencephaly	51 (28.5)	5 (45.5)	NS
Short stature	12 (6.7)	1 (9.1)	NS
Learning disability	24 (13.4)	2 (18.2)	NS
Special education	3 (1.7)	0 (0)	NA
Mental retardation	12 (6.7)	4 (36.4)	0.0006
Speaking problems	7 (3.9)	0 (0)	NA
Behavior problems	5 (2.8)	0 (0)	NA
Epilepsy	10 5.6)	0 (0)	NA
Other	38 (21.2)	4 (36,4)	NS

Patient	Intragenic mutation	Missense mutation	*P*‐value
Number	161	18	
Café‐au‐lait	150 (93.2)	17 (94.4)	NS
Freckles	115 (71.4)	13 (72.2)	NS
Lisch nodules	77 (47.8)	7 (38.9)	NS
Neurofibromas	110 (68.3)	9 (50)	NS
Plexiform neurofibromas	28 (17.4)	1 (5.6)	NS
Optic glioma	31 (19.3)	3 (16.7)	NS
Osseous lesion	8 (5)	0 (0)	NA
NF1 Familiar	51 (31.7)	5 (27.8)	NS
No minor feature	58 (36)	8 (44.4)	NS
Tumors	24 (14.9)	4 (22.2)	NS
Scoliosis	26 (16.1)	2 (11.1)	NS
Macroencephaly	48 (29.8)	3 (16.7)	NS
Short stature	11 (6.8)	1 (5.6)	NS
Learning disability	21 (13)	3 (16.7)	NS
Special education	3 (1.9)	0 (0)	NA
Mental retardation	11 (6.8)	1 (5.6)	NS
Speaking problems	7 (4.3)	0 (0)	NA
Behavior problems	5 (3.1)	0 (0)	NA
Epilepsy	9 (5.6)	1 (5.6)	NS
Other	32 (19.9)	6 (33.3)	NS

Parentheses values are expressed in percent. NS, nonstatistical significant; NA, not available.

## Discussion

We report the mutational spectrum of Italian NF1 patients referred to our center between 2003 and 2014. Thus far, no national NF1 register has been instituted in Italy, and few papers have described Italian genetic records (Origone et al. 2002; De Luca et al. 2004, 2007), although the findings of an Italian mortality study were consistent with those performed in other industrialized countries (Zöller et al. 1995; Rasmussen et al. 2001).

The detection rate obtained following our screening protocol changed depending on the technology applied. Diagnostic protocols evolved, beginning in 2003 with a canonical DNA‐based approach of DHPLC and DNA sequence screening. In 2008, we introduced MLPA analysis to detect gross duplications and deletions in the NF1 gene. Beginning in January 2013, an integrated DNA‐RNA protocol was routinely used.

These new technical approaches allowed us to improve our starting detection rate from 58% in 2003–2008 to 75% in 2008–2013 (MLPA introduction), and finally to 87% with the introduction of RNA analysis.

Our DNA detection rate is slightly lower than other reports based on DNA analysis, such as Griffiths et al. (2007) (78%) and Van Minkelen et al. (2014) (81%), possibly due to the large size of the Dutch patient cohort screened in those studies and the diversity of the techniques used. Indeed, higher detection rates were possible only with a multistep protocol, when analyses of DNA and RNA were integrated (Nemethova et al. 2013; Sabbagh et al. 2013) 95% was reached by Messiaen et al. (2000) through a comprehensive protocol including cytogenetic analysis, MLPA, RNA analysis and DNA sequencing. In our experience, RNA analysis resulted in a decrease in the misclassification of missense and silent mutations facilitated the observation of new splicing mutations in deep intronic regions, and decreased the time spent by the analyst.

Among the 513 unrelated cases, our diagnostic protocols (i.e., both DNA and RNA analysis) revealed 225 distinct pathogenic mutations in the NF1 gene. As expected based on the literature, small changes represent 92% of mutations (206/225) and span the entire gene without any hot region (Fig. 4). The residual 8% was composed of gross deletions (17/19) or duplications of exons (2/19).

By searching two main genetic databases (HGMD and LOVD) for our 225 distinct mutations, we found that 126 were novel, including 110 small changes and 16 aberrant deletions/duplications.

The description of new mutations is important for NF1 patients due the high mutational rate of the gene and the high phenotypic variability of the disease, even among members of the same family. Thus, reporting new mutations could help physicians and genetic counselors with the diagnosis and treatment of NF1 patients worldwide.

The 126 new mutations were nearly all present in 1 unrelated patient (“private”), and 64% of the mutations were de novo; 86% of the patients with these novel mutations fulfilled NIH criteria and were characterized by high phenotypic variability.

Most of the novel mutations were small changes (87.5%) and were predominantly small deletions/insertions (44%). Splicing mutations represented the second most prominent class of mutations (27/126 – 21%), in agreement with previous reports (Griffiths et al. 2007; van Minkelen et al. 2014). (Messiaen et al. 2000; Wimmer et al. 2007; Sabbagh et al. 2013). However, RNA approach usually identify 30–34% of splicing alterations (Messiaen et al. 2000; Wimmer et al. 2007; Sabbagh et al. 2013).

We observed a higher frequency of missense variation than previously reported in literature (12 vs. 7.4–9.8%) (Messiaen et al. 2000; van Minkelen et al. 2014) This difference could be due to the cohort of novel mutations or our DNA‐based protocol; RNA studies reveal that new missense and new silent mutations could alter correct splicing if they fall at consensus sites (Pros et al. 2008). Only 2 of the 15 novel missense alterations were identified by the RNA approach.

In our patients, four missense mutations screened by DNA are predicted to be splicing mutations influencing correct splicing form. Further studies of RNA will confirm any eventual splicing effects of these missense mutations on NF1 transcripts that were underestimated by a DNA‐based approach. Sabbagh and colleagues verified that approximately 31.1% of missense mutations actually disturb NF1 transcript splicing (Sabbagh et al. 2013).

The majority of alterations resulted in the formation of frameshifts and truncated proteins that could also be induced by splicing alterations.

We found a preferential alteration in the conserved nucleotides of the consensus splice site. Splicing alteration at the donor (5′) and acceptor (3′) sites usually causes exon skipping. Donor (5′) sites were compromised in 13 of the 21 (62%) splicing mutations, as predicted by bioinformatics tools. Splicing mutations were probably underestimated due to the nature of the DNA analysis used for the majority of the patients included in our analysis. Only 25 of the 126 mutations were detected by the MLPA‐RNA sequencing approach. Moreover, we detected 16 novel gross alterations.

The description of mutations detected in unrelated patients and their correlation with phenotypic reports is very important for this multisystemic disease with high expression variability. The splicing mutation c.1466A>G is the most frequent alteration among unrelated patients in our database. This mutation has been described in the literature (Messiaen et al. 1999; Osborn and Upadhyaya 1999) and is found 34 times in LOVD database. Interestingly, the 9 patients with this alteration showed variable phenotypes (Table 1). In particular, NF1_46 did not fulfill the NIH criteria for diagnosis (presenting with only 3 neurofibromas), indicating the importance of cataloging novel mutations and of sharing such information in databases such as LOVD.

No obvious genotype–phenotype correlation has been demonstrated in NF1 patients. To date only two correlations of clinical significance have been reported: del AAT in exon 22 is associated with absence of neurofibromas (described above; Upadhyaya et al. 2007) and the “NF1 microdeletion phenotype” (Mensink et al. 2006; Mautner et al. 2010; Pasmant et al. 2010). Such patients have a higher incidence of intellectual disability (mental retardation), developmental delay, dysmorphic facial features, the earlier appearance of cutaneous neurofibromas, and connective tissue abnormalities. Recently, Pinna et al. (2014) published a mild phenotype linked to changes in the amino acid residue 1809. This substitution correlated with a mild phenotype characterized by CALs and skinfold freckling, but lacked discrete cutaneous or PN, LNs, and typical NF1 osseous lesions. In this Italian cohort, we describe 11 cases with a type 1 microdeletion (Kluwe et al. 2004) and two type 3 microdeletions (Bengesser et al. 2010), but no type 2 microdeletions (Kehrer‐Sawatzki et al. 2004). Two patients confirmed that a mild phenotype was correlated with the small deletion c.2970_2972delAAT and the missense alteration in the amino acid residue 1809 (p.Arg1809Cys).

We performed statistical analyses in 207 NF1 patients with reviewed clinical data. No novel genotype–phenotype correlation was discovered (Table 5). As expected, adults fulfilling clinical criteria displayed more neurofibromas and LNs, whereas children displayed more CALs and lentigo.

Statistical analysis was significant for only microdeletion type 1 and mental retardation.

Our data reveal important considerations for patients who did not fulfill clinical criteria at the time of diagnosis. Among the 513 unrelated individuals investigated, 159 did not fulfill the criteria described at the NIH conference in 1988. In addition, 36 had mutations in the NF1 gene.

Eighty‐nine of the 159 patients were children of less than 12 years of age (56%). In children, clinical diagnosis is problematic because 46% of sporadic cases fail to meet NIH criteria by the age of 1 year (DeBella et al. 2000). Many features of NF1 increase in frequency with age: nearly all by the age of 12 years and all by 20 years. In fact, the majority of these patients not fulfilling the NIH criteria display CALs and minor features (Table S1). Adults that did not fulfill NIH criteria showed neurofibromas, CALs or both, but fell below the threshold for NIH diagnosis (Table S1). 11% of these adults (6 on 56/159) were oligosymptomatic cases with *NF1* mutations (Table 1).

Adult patients with *NF1* mutations not fulfilling the NIH criteria have been described and have distinct clinical features: (a) segmental forms of NF1, probably due to mosaic *NF1* mutations; (b) spinal neurofibromatosis with extensive, symmetrical multiple tumors involving large regions of the spine and few, if any, cutaneous manifestations; (c) late‐onset neurofibromatosis as defined by Riccardi (1992) with only neurofibromas beginning in the 3rd decade; and (d) optic glioma with few cutaneous manifestations (Buske et al. 1999). Two of our six cases had late‐onset NF1 (NF1_46 and NF1_553), one optic glioma, and few cutaneous manifestations (NF_355). Three cases had different and new clinical features: a girl had a glioma outside the optic nerve and less than 6 CALs (NF1_212), and there were two cases of learning disability and a few neurofibromas or CALs (NF1_19 and NF1_552).

The accuracy of diagnostic criteria is critical for autosomal dominant diseases, which are characterized by variable expression and a predisposition to cancer. Recent papers suggest that the NIH diagnostic criteria require reexamination (Burkitt Wright et al. 2013; Gutmann 2014; Tadini et al. 2014; Epstein et al. 2015). Our data suggest that clinical criteria such as the presence of glioma or learning disability as well as cutaneous features should be formally assessed and considered when deciding whether to perform molecular analysis, although further observations are needed to establish their role as potential diagnostic criteria.

## Conflict of Interest

The authors declare no conflicts of interest.

## Supporting information


**Table S1**. Not fulfilling NIH criterion clinical data.

**Table S2.** Novel small mutations.

**Table S3**. Mutations in NF1 protein domain.

**Table S4.** Small mutations described in literature.
