# Supplementary material for: 126 novel mutations in Italian patients with neurofibromatosis type 1
Source: Mol Genet Genomic Med. 2015 Jul 7;3(6):513–25. doi: 10.1002/mgg3.161 (PMC4694136; doi:10.1002/mgg3.161)
Supplement: Supplementary file 1 — Table S1. Not fulfilling NIH criterion clinical data. [file MGG3-3-513-s001.docx]

**Supplementary Table 1**. *Not fulfilling NIH criterion clinical data*.

| **Patient** | **Under 18 year** | **Over 18 year** |
| --- | --- | --- |
| **Number** | **101** | **52** |
| Café-au-lait | 75 (74,3) | 7 (13,5) |
| Freckls | 2 (2) | 1 (1,9) |
| Lisch nodules | 0 (0) | 0 (0) |
| Neurofibromas | 4 (4) | 25 (48,1) |
| Plexiphorm neurofibromas | 1 (1) | 0 (0) |
| Opthic glioma | 2 (2) | 2 (3,8) |
| Osseous lesion | 0 (0) | 0 (0) |
| NF1 Familiar | 3 (3) | 3 (5,8) |
| No minor feature | 50 (49,5) | 23 (44,2) |
| Tumors | 0 (0) | 6 (11,5) |
| Scoliosis | 1 (1) | 2 (3,8) |
| Macroencephaly | 4 (4) | 0 (0) |
| Short stature | 0 (0) | 0 (0) |
| Learning disability | 16 (15,8) | 5 (9,6) |
| Special education | 0 (0) | 0 (0) |
| Mental retardation | 2 (2) | 0 (0) |
| Speaking problems | 0 (0) | 0 (0) |
| Behavior problems | 0 (0) | 0 (0) |
| Epilepsy | 2 (2) | 0 (0) |
| Other | 26 (25,7) | 7 (13,5) |

*Percent* ().
