# Supplementary material for: 126 novel mutations in Italian patients with neurofibromatosis type 1
Source: Mol Genet Genomic Med. 2015 Jul 7;3(6):513–25. doi: 10.1002/mgg3.161 (PMC4694136; doi:10.1002/mgg3.161)
Supplement: Supplementary file 2 — Table S2. Novel small mutations. [file MGG3-3-513-s002.docx]

**Supplementary Table 2**. Novel small mutations.

| **Patient** | **Frequency** | **Position** | **DNA Mutation** | **RNA Mutation** | **Protein Prediction** | **Effect** |
| --- | --- | --- | --- | --- | --- | --- |
| NF1_239 | 1 | E1 | c.21_22delGG |  | p.(Glu8Metfs*29) | splicing |
| NF1_317 | 1 | E2 | c.99A>G |  | p.(?) | splicing |
| NF1_428 | 1 | E2 | c.185dupT |  | p.(Leu62Phefs*5) | Ins |
| **NF1_623** | 1 | E2 | c.200dupA | r.200dupA | p.Asn67Lysfs*10 | dup |
| NF1_177-483 | 2 | E3 | c.240_243delTCTC |  | p.(Gln83*) | del |
| NF1_314 | 1 | E3 | c. 247delCinsGAGA |  | p.(Gln83delinsGluLys) | del/ins |
| NF1_499 | 1 | E3 | c. 252delG |  | p.(Ile85Leufs*18) | del |
| NF1_136 | 1 | IVS3 | c.288+1delG |  | p.(?) | splicing |
| NF1_354 | 1 | E4 | c. 479G>T |  | p.(Arg160Met) | splicing |
| NF1_330 | 1 | E5 | c. 529_530insGA |  | p.(Ile177Argfs*2) | ins |
| **NF1_644** | 1 | E6 | c.615_616delGAinsAT | r.587_654del68 | p.Glu196Glyfs*12 | splicing |
| NF1_82 | 1 | E6 | c.647_649delTGG |  | p.(Leu216_Lys217delinsGln) | del |
| NF1_384 | 1 | E6 | c.652_653delAAinsG |  | p.(Lys218Glyfs*7) | del/ins |
| NF1_228 | 1 | E8 | c. 755T>A |  | p.(Leu252*) | nonsense |
| NF1_333 | 1 | E9 | c.953_956delAAAG |  | p.(Glu318Valfs*57) | del |
| NF1_355 | 1 | IVS9 | c.1062+113A>G |  | p.(Asn355Valfs*12) | splicing |
| NF1_173 | 1 | IVS9 | c.1063-2A>C |  | p.(?) | splicing |
| NF1_219 | 1 | E10 | c.1105C>T |  | p.(Gln369*) | nonsense |
| NF1_195 | 1 | E10 | c.1140delT |  | p.(Val381Phefs*6) | del |
| NF1_111 | 1 | E10 | c.1174 C>T |  | p.(Gln392*) | nonsense |
| NF1_385 | 1 | E10 | c.1180dupT |  | p.(Lys395*) | ins |
| NF1_341 | 1 | E11 | c.1260dupT |  | p.(Ser421Phefs*8) | ins |
| **NF1_655** | 1 | E13 | c.1453G>T | r.1453G>U | p.Glu485* | nonsense |
| **NF1_869** | 1 | E13 | c.1467dupT | r.1467dupU | p.Lys490* | nonsense |
| **NF1_710** | 1 | E13 | c.1496T>G | r.1496U>G | p.Leu499Arg | missense |
| NF1_269 | 1 | E13 | c.1520delT |  | p.(Leu507Cysfs*19) | del |
| NF1_144 | 1 | E14 | c.1552_1553delAC |  | p.(Thr518Profs*39) | del |
| NF1_193 | 1 | E14 | c.1555delC |  | p.(Gln519Lysfs*7) | del |
| NF1_254 | 1 | E14 | c.1603 C>T |  | p.(Gln535*) | nonsense |
| NF1_434 | 1 | E14 | c.1634C>A |  | p.(Ala545Glu) | missense |
| NF1_135 | 1 | E15 | c.1713_1716delGGGA |  | p.(Trp571*) | del |
| NF1_372 | 1 | IVS15 | c.1722-2A>T |  | p.(?) | splicing |
| NF1_208 | 1 | E16 | c.1722C>G |  | p.(Ser574Arg) | missense |
| **NF1_624** | 1 | E16 | c.1845G>A | r.[1642_1845del204;1722_1845del124] | p.[Ala548_Lys615del; Ser575Argfs*15] | splicing |
| NF1_220 | 1 | E18 | c.2019delC |  | p.(Cys673*) | del |
| NF1_450 | 1 | E18 | c.2067_2070delGGCC |  | p.(Ala690Cysfs*57) | del |
| NF1_349 | 1 | E18 | c.2084_2085delTG |  | p.(Trp696Glufs*3) | del |
| NF1_488 | 1 | E18 | c.2205T>G |  | p.(Tyr735*) | nonsense |
| NF1_171 | 1 | E19 | c.2325G>C |  | p.(Glu775Asp) | splicing |
| **NF1_805** | 1 | IVS19 | c.2325+2T>G | r.2252_2325del74 | p.Arg752Leufs*17 | splicing |
| NF1_558 | 1 | E20 | c.2356delC |  | p.(Gln786Lysfs*5) | del |
| NF1_416 | 1 | IVS20 | c.2410-18C>G | r.2410-18ins18 | p.Gln803fs*23 | splicing |
| NF1_507 | 1 | E21 | c.2492_2493dupCA | r.2492_2493dupCA | p.Asp832Glnfs*10 | dup |
| NF1_441 | 1 | E21 | c.2846delG |  | p.(Gly949Aspfs*5) | del |
| **NF1_630** | 1 | E21 | c.2850G>A | r.2618_2850del233 | p.Lys874Phefs*4 | splicing |
| NF1_487 | 1 | E22 | c.2870delA |  | p.(Asn957Ilefs*5) | del |
| **NF1_743** | 1 | IVS22 | c.2990+5G>C | r.2851_2990del140 | p.Leu952Cysfs*22 | splicing |
| **NF1_755** | 1 | E23 | c.3037delA | r.3037delA | p.Thr1013Argfs*6 | del |
| NF1_244 | 1 | E25 | c.3278T>A |  | p.(Val1093Glu) | missense |
| NF1_407 | 1 | E26 | c.3335delA |  | p.(Asn1112Thrfs*3) | del |
| NF1_8-399/120 | 2 | E26 | c.3337delC |  | p.(Leu1113Phefs*2) | del |
| NF1_459 | 1 | E26 | c.3384_3390delTGGCAGG |  | p.(Gly1129Asnfs*11) | del |
| NF1_89 | 1 | E27 | c.3523A>G |  | p.(Thr1175Ala) | missense |
| NF1_394 | 1 | E27 | c.3586C>G |  | p.(Leu1196Val) | missense |
| NF1_117 | 1 | E27 | c.3644T>G |  | p.(Met1215Arg) | missense |
| NF1_216 | 1 | E28 | c.3844delA |  | p.(Ser1282Valfs*3) | del |
| **NF1_556** | 1 | E28 | c.3748C>A | r.3709_3749del41 | p.Asp1237Alafs*13 | splicing |
| **NF1_614** | 1 | E28 | c.3826_3828delCGAinsTACT | r.3826_3828delCGAinsUACU | p.Arg1276Tyrfs*8 | del/ins |
| NF1_429-**705** | 2 | E29 | c.3941G>A |  | p.(Trp1314*) | nonsense |
| NF1_104 | 1 | IV 29 | c.3974+1G>A |  | p.(?) | splicing |
| NF1_225 | 1 | IVS29 | c.3974+2T>G |  | p.(?) | splicing |
| NF1_328 | 1 | E30 | c.3995delT |  | p.(Glu1333Argfs*10) | del |
| NF1_99 | 1 | E31 | c.4118_4119delGC |  | p.(Ser1373Thrfs*29) | del |
| **NF1_857** | 1 | E31 | c.4154delG | r.4154delG | p.Gly1385Glufs*22 | del |
| NF1_339 | 1 | E31 | c.4236_4237delTAinsGATT |  | p.(Arg1413Ilefs*7) | del/ins |
| NF1_408 | 1 | IVS31 | c.4269+2T>G |  | p.(?) | splicing |
| NF1_153 | 1 | E32 | c.4301delT |  | p.Phe1434Serfs*14 | del |
| NF1_70 | 1 | E33 | c.4381A>G |  | p.(Ile1461Val) | missense |
| **NF1_733** | 1 | E33 | c.4402_4406delAGTGA | r.4402_4406delAGUGA | p.Ser1468Cysfs*5 | del |
| **NF1_737** | 1 | E33 | c.4423dupC | r.4423dupC | p.Leu1475Profs*6 | dup |
| NF1_30 | 1 | E34 | c.4606dupA |  | p.(Thr1536Asnfs*7) | ins |
| NF1_156 F | 1 | E36 | c.4830_4844del |  | p.(Lys1611_Ala1615del) | del |
| NF1_424 | 1 | E36 | c.4859T>C |  | p.(Ile1620Thr) | missense |
| NF1_305 | 1 | E36 | c.4867G>C |  | p.(Asp1623His) | missense |
| NF1_403 | 1 | E36 | c.5047A>T |  | p.(Lys1683*) | nonsense |
| NF1_32 | 1 | E36 | c.5154_5158dupATTC |  | p.(His1720Ilefs*17) | ins |
| NF1_41 | 1 | E36 | c.5162A>G |  | p.(Asn1721Ser) | missense |
| NF1_506 | 1 | E37 | c.5472insT |  | p.(Arg1825Serfs*16) | ins |
| NF1_49 | 1 | E39 | c.5890G>T |  | p.(Glu1964*) | nonsense |
| NF1_360 | 1 | E39 | c.5927G>A |  | p.(Trp1976*) | nonsense |
| NF1_319 | 1 | E39 | c.5943G>T |  | p.(Gln1981His) | splicing |
| NF1_206 | 1 | IVS39 | c.5944-1G>T |  | p.(?) | splicing |
| NF1_197 | 1 | E40 | c.6084G>C |  | p.(Lys2028Asn) | missense |
| **NF1_571** | 1 | E41 | c.6311T>C | r.6311U>C | p.(Leu2104Pro) | missense |
| NF1_112 | 1 | E41 | c.6347C>G |  | p.(Ser2116*) | nonsense |
| **NF1_536** | 1 | E42 | c.6477delC | r.6477delC | p.Ser2160Valfs*19 | del |
| NF1_6 | 1 | E44 | c.6642-?_6756+?dup140 |  | p.(?) | dup |
| NF1_7 | 1 | E44 | c.6688delG |  | p.(Val2230Serfs*14) | del |
| NF1_393 | 1 | E44 | c.6692_6693delTT |  | p. Phe2231Trpfs*4 | del |
| **NF1_603** | 1 | E45 | c.6760delC | r.6760delC | p.Glu2255Argfs*4 | del |
| **NF1_736** | 1 | E45 | c.6790dupT | r.6790dupU | p.Tyr2264Leufs*22 | dup |
| **NF1_807** | 1 | E46 | c.6881delT | r.6881delU | p.Leu2294Profs*4 | del |
| NF1_2 | 1 | E46 | c.6907C>T |  | p.(Gln2303*) | nonsense |
| NF1_23 | 1 | E46 | c.6938G>A |  | p.(Gly2313Asp) | missense |
| NF1_29 | 1 | IVS46 | c.6999+1G>C |  | p.(?) | splicing |
| NF1_340 | 1 | IVS46 | c.6999 +1G>T |  | p.(?) | splicing |
| NF1_460 | 1 | IVS46 | c.7000-1G>C |  | p.(?) | splicing |
| NF1_231-313 | 2 | IVS47 | c.7126+2T>G |  | p.(?) | splicing |
| NF1_553 | 1 | E48 | c.7240A>T |  | p.(Ser2414Cys) | missense |
| NF1_79 | 1 | E49 | c.7309dupA |  | p.(Arg2437Lysfs*8) | ins |
| **NF1_702** | 1 | E50 | c.7422dupC | r.7422dupC | p.Ser2475Leufs*6 | ins |
| NF1_78 | 1 | E50 | c.7425_7426insTCTC |  | p.(Pro2476Serfs*6) | ins |
| NF1_463 | 1 | E50 | c.7519delC |  | p.(Gln2507Asnfs*20) | del |
| NF1_404 | 1 | E51 | c.7580dupA |  | p.(Ser2528Ilefs*7) | ins |
| NF1_201 | 1 | E51 | c.7619C>G |  | p.(Ser2540*) | nonsense |
| NF1_322 | 1 | E52 | c.7793T>G |  | p.(Leu2598Arg ) | missense |
| NF1_210 | 1 | IVS52 | c.7806+1G>A |  | p.(?) | splicing |
| NF1_169 | 1 | IVS53 | c.7907+1G>T |  | p.(?) | splicing |
| NF1_552 | 1 | E54 | c.7994A>G |  | p.(Gln2665Arg ) | missense |
| NF1_202 | 1 | E57 | c.8111delC |  | p.(Pro2704Glnfs*14) | del |
|  |  |  |  |  |  |  |

E= exon; IVS=intron; Del=deletion; Ins=insertion; Dup=duplication; **bold number=**patients screened through RNA approach
