# Supplementary material for: 126 novel mutations in Italian patients with neurofibromatosis type 1
Source: Mol Genet Genomic Med. 2015 Jul 7;3(6):513–25. doi: 10.1002/mgg3.161 (PMC4694136; doi:10.1002/mgg3.161)
Supplement: Supplementary file 3 — Table S3. Mutations in NF1 protein domain. [file MGG3-3-513-s003.docx]

**Supplementary Table 3**. *Mutations in NF1 protein domain.*

| **Main Protein Domain** | **Internal Domain** | **Aminoacid Involved** | **N° of variation** |
| --- | --- | --- | --- |
| **CSRD** |  | 543-909 | 14 |
| **GRD** |  | 1168-1530 | 18 |
| **LZD** |  | 1543-1550 | 0 |
| **CTD** |  | 2260-2818 | 21 |
|  | NLS | 2534-2550 | 1 |
|  | TRS | 2549-2556 | 0 |

*Cystein Serin Rich Domain (CSRD); Gap Region Domain (GAP); Leucine Zipe Domain (LZD); C-Terminal Domain (CTD);* *Nuclear Localization Site (NLS) and the Tyrosine Kinase Recognition sites (TRS).*
