# Supplementary material for: 126 novel mutations in Italian patients with neurofibromatosis type 1
Source: Mol Genet Genomic Med. 2015 Jul 7;3(6):513–25. doi: 10.1002/mgg3.161 (PMC4694136; doi:10.1002/mgg3.161)
Supplement: Supplementary file 4 — Table S4. Small mutations described in literature. [file MGG3-3-513-s004.docx]

**Supplementary Table 4.** Small mutations described in literature.

| **Patient** | **Frequency** | **Position** | **DNA Mutation** | | **RNA Mutation** | **Protein Prediction** | **Reference** | **Effect** |
| --- | --- | --- | --- | --- | --- | --- | --- | --- |
| NF1_446 | 1 | E1 | c.27G>A | |  | p.(Trp9*) | LOVD | nonsense |
| **NF1_657** | 1 | E1 | c.47G>C | | r.47G>C | p.Arg16Pro | Baralle et al. 2005 | missense |
| NF1_34 | 1 | E3 | c.236T>G | |  | p.(Leu79*) | LOVD | nonsense |
| NF1_469 | 1 | E3 | c.247C>T | |  | p.(Gln83*) | Osborn and Upadhyaya 1999 | nonsense |
| NF1_121-235 | 2 | E5 | c.484C>T | |  | p.(Gln162*) | Keburi et al. 2008 | nonsense |
| NF1_22-232-323-**596** | 4 | E5 | c. 495_498delTGTT | | r.495_498delUGUU | p.Thr166fs*11 | Osborn et al. 1999 | del |
| NF1_412 | 1 | E5 | c. 499_502delTGTT | |  | p.(Thr167Glnfs*9) | Ars et al. 20003; Toliat 2000 | del |
| **NF1_694** | 1 | E5 | c.541C>T | | r.541C>U | p.Glu181* | Griffiths 2007 | nonsense |
| NF1_66 -74 -125-**738** | 4 | E5 | c.574C>T | | r.574C>U | p.Arg192* | Toliat et al. 2000; Fashold 2000 | nonsense |
| NF1_28 | 1 | IVS5 | c.586+5G>A | |  | p.(Leu161fs*2) | Ars et al. 2000 | splicing |
| NF1_461 | 1 | E6 | c.649delG | |  | p.(Glu217Lysfs*8) | Pros et al. 2008 | del |
| NF1_310 | 1 | E7 | c.663G>A | |  | p.(Trp221*) | LOVD | nonsense |
| NF1_106 | 1 | E8 | c.809A>C | |  | p.(Gln270Pro) | LOVD | missense |
| NF1_87 | 1 | E9 | c.910C>T | |  | p.(Arg304*) | Hoffmeyer et al. 1998; Fahsold et al. 2000; Upadhyaya et al. 2008 | nonsense |
| NF1_179 | 1 | E9 | c.1019_1020delCT | |  | p.(Ser340Cysfs*12) | Upadhyaya et al. 1997; Ars et al. 2003 | nonsense |
| NF1_312 | 1 | E9 | c.1021_1022delGT | |  | p.(Val341Hisfs*11) | De Luca et al. 2004 | nonsense |
| NF1_316 | 1 | E11 | c.1246C>T | |  | p.(Arg416*) | Osborn et al. 1999; Fashold et al. 2000 | nonsense |
| NF1_462 | 1 | IVS11 | c.1260+1G>A | |  | p.(Ser421fs) | Eisenbarth et a.l 2000 | splicing |
| NF1_447 | 1 | IVS11 | c.1260+1604A>G | |  | p.(Ser421Leufs*4) | Valero et.al. 2011 | splicing |
| NF1_241-281-356-425 | 4 | E12 | c.1318C>T | |  | p.(Arg440*) | Heim et al. 1995; Fahsold et al. 2000 | nonsense |
| NF1_280-392 | 2 | E12 | c.1381C>T | |  | p.(Arg461*) | Fahsold et al. 2000; Messian et al.2000 | nonsense |
| NF1_168 | 1 | E12 | c.1392+1G>T | |  | p.(Ser421_Pro464del) | Sabbagh et al. 2013 | splicing |
| NF1_259 | 1 | E13 | c.1399insA | |  | p.(Thr467Asnfs*3) | Ars et al. 2003 | ins |
| NF1_46-48-157-199-229-245- 279-**712-775** | 9 | E13 | c.1466A>G | | r.1466A>G | p.Tyr489* | Osborn et al.1999; Messian et al. 1999; Ars et al.2000; Pros et al. 2008 | splicing |
| NF1_100-218 | 2 | E14 | c.1541_1542delAG | |  | p.(Gln514fs*21) | Robinson et al. 1996 | del |
| NF1_203 | 1 | E14 | c.1542delG | |  | p.(Lys514fs*10) | Kluwe et al. 2002 | del |
| **NF1_720** | 1 | E15 | c.1658A>G | | r.1658A>G | p.Hys553Arg | Li et al. 2007 | missense |
| NF1_370-432 | 2 | IVS15 | c.1721+3A>G | |  | p.(Ala548fs) | Purandare et al. 1994 | splicing |
| NF1_162-318-423 | 3 | E16 | c.1756_1759delACTA | |  | p.(Thr586fs*17) | Park et al.1998 | del |
| **NF1_658** | 1 | E16 | c.1830_1833delTCTT | | r.1830_1833delUCUU | p.Leu612Lysfs*18 | Bottillo et al. 2009 | del |
| NF1_522 | 1 | IVS16 | c.1846+1G>A | |  | p.(?) | Fang et al. 2001 | splicing |
| **NF1_692** | 1 | E17 | c.1885G>A | | r.1885del41 | p.GLn616fs | Gasparini et al. 1996; Pros et al. 2008 | splicing |
| NF1_304-**633-663** | 3 | E18 | c.2033dupC | | r.2033dupC | p.Ile679Aspfs*21 | Heim et al. 1995; Fahsold et al. 2000 | ins |
| NF1_421-**696** | 2 | E18 | c.2041C>T | | r.2041 C>U | p.Arg681* | Ars et al. 2000 | nonsense |
| NF1_212 | 1 | E19 | c.2291T>C | |  | p.(Leu763Pro) | Fahsold et al. 2000 | missense |
| NF1_272 | 1 | IVS20 | c.2409+1G>A | |  | p.(?) | Lee et al. 2006 | splicing |
| NF1_344 | 1 | IVS20 | c.2409+1G>T | |  | p.(?) | Lee et al. 2006 | splicing |
| NF1_289 | 1 | E21 | c.2541T>C | |  | p.(Leu847Pro) | Messian 1998; Fahsold et al.2000 | missense |
| NF1_471 | 1 | IVS21 | c.2990+1G>C | |  | p.Leu952fs | Sabbagh et al. 2013 | splicing |
| NF1_629 | 1 | E22 | c.2970_2972delAAT | |  | p.(Met991del) | Shen et al. 1993; Upadhyaya et al. 2007 | del |
| NF1_448 | 1 | IVS22 | c.2991-1G>A | |  | p.(?) | Perrin et al. 1996 | splicing inframe |
| NF1_435 | 1 | IVS22 | c.2991-2A>G | |  | p.(Tyr998_Arg1038del) | Osborne et al. 1999 | splicing in frame |
| **NF1_535** | 1 | IVS22 | c.2991-2A>C | | r.2991_3113del123 | p.Tyr998_Arg1038del | LOVD | splicing in frame |
| NF1_242 | 1 | E23 | c.3047_3048delGT | |  | p.(Cys1016fs*3) | Lee et al.2006 | del |
| **NF1_587** | 1 | E24 | c.3163C>T | | r.3163C>U | p.Gln1055* | Mattock et al.2004 | nonsense |
| NF1_298 | 1 | IVS24 | c.3198-2A>G | |  | p.(Asp1067fs*20) | Upadhyaya et al. 2008 | splicing |
| NF1_311 | 1 | E25 | c.3311T>G | |  | p.(Leu1104Arg | LOVD | missense |
| NF1_85 | 1 | E25 | c.3449C>G | |  | p.(Ser1150*) | LOVD | nonsense |
| NF1_3-**739** | 2 | E25 | c.3456_3460delACTC | | r.3456_3460delACUC | p.Leu1153fs*3 | Upadhyaya et al. 1997 | del |
| NF1_559 | 1 | IVS25 | c.3496+2T>C | |  | p.(?) | Klose et al. 1998. | splicing |
| NF1_438 | 1 | E27 | c.3520C>T | |  | p.(Gln1174*) | Messiaen et al. 2000 | nonsense |
| NF1_84 | 1 | E27 | c.3525_3256delAA |  | | p.(Arg1176fs) | Fahsold et al. 2000 | del |
| NF1_54 | 1 | E27 | c.3610T>C | |  | p.(Arg1204Trp) | Ars at al. 2000 | missense |
| NF1_45 | 1 | IVS27 | c.3708+1G>C | |  | p.(?) | LOVD | splicing |
| NF1_454 | 1 | E28 | c.3721C>T | |  | p.(Arg1241*) | Fahsold et al. 2000 | nonsense |
| NF1_509 | 1 | E28 | c.3739_3742delTGTT | |  | p.(Phe1247Ilefs*18) | Fahsold et al. 2000 | del |
| NF1_200-302-398-**611** | 4 | E28 | c.3826C>T | |  | p.(Arg1276*) | Heim et al. 1995 | nonsense |
| NF1_167 | 1 | E28 | c.3847delA | |  | p.(Ile1284*) | De Luca et al. 2004 | del |
| NF1_19-**700-688** | 3 | E29 | c.3916C>T | | r. 3916 C>U | p.Arg1306* | Park et al. 1998 | nonsense |
| NF1_383 | 1 | IVS29 | c.3975-2A>G | |  | p.(Arg1325fs) | Upadhyaya et al. 1997; Fahsold et al. 2000; Messian et al. 2000 | splicing |
| NF1_110 | 1 | E30 | c.4054delA | |  | p.(Ser1352fs*3) | LOVD | del |
| NF1_134-204-303-308 | 4 | E30 | c.4084C>T | |  | p.(Arg1362*) | Upadhyaya et al. 1997; Fahsold et al. 2000; Messian et al. 2000 | nonsense |
| NF1_375 | 1 | E31 | c.4172G>C | |  | p.(Arg1391Thr ) | LOVD | missense |
| **NF1_677** | 1 | E31 | c.4267A>G | | r..4267A>G | p.Lys1423Glu | Li et al. 1992 | missense |
| NF1_327 | 1 | E33 | c.4440delC | |  | p.(Asp1480Glufs*73) | LOVD | del |
| NF1_283 | 1 | IVS33 | c.4515-1G>A | |  | p.(?) | Mattocks et al. 2004 | splicing |
| NF1_528 -170 | 2 | E34 | c.4537C>T | |  | p.(Arg1513*) | Side et al. 1997; Fahsold et al. 2000 | nonsense |
| NF1_273 | 1 | E34 | c.4630delA | |  | p.(Thr1544Profs*9) | Upadhyaya et al. 1995 | del |
| NF1_55 | 1 | IVS36 | c.5206-2A>G | |  | p.(?) | Girondon-Boulandet et al. 2000 | splicing |
| NF1_391 | 1 | E37 | c.5234C>G | |  | p.(Ser1745*) | Upadhyaya et al. 2008 | nonsense |
| NF1_222-271-276-306 | 4 | E37 | c.5242C>T | |  | p.(Arg1748*) | Valero et al.1994 | nonsense |
| NF1_379 | 1 | E237 | c.5353C>T | |  | p(.Gln1785*) | Fahasold et al. 2000 | nonsense |
| NF1_369 | 1 | E37 | c.5425C>T | |  | p.(Arg1809Cys) | Syrbe et al. 2007 | missense |
| NF1_213 | 1 | E237 | c.5546G>A | |  | p.(Gly1737fs ) | Ars et al. 2003 | splicing |
| NF1_502 | 1 | IVS37 | c.5546+5G>C | |  | p.(?) | Lee et al. 2006 | splicing |
| NF1_433 | 1 | E38 | c.5624C>G | |  | p.(Ser1875*) | LOVD | nonsense |
| NF1_439 | 1 | E38 | c.5681T>G | | r.5681U>G | p.Leu1894Pro | LOVD | missense |
| NF1_289 | 1 | E39 | c.5839C>T | |  | p.(Arg1947*) | Cawthon et al. 1990 | nonsense |
| NF1_252-284-294 | 3 | E39 | c.5896C>T | |  | p.(Gln1966*) | Messiaen et al. 2000 | nonsense |
| NF1_531 | 1 | IVS41 | c.6364+2T>A | |  | p.(?) | LOVD | splicing |
| **NF1-541** | 1 | IVS43 | c.6641+1G>A | | r.6580_6641del62 | p.Ala2194fs | De Luca et al. 2004 | splicing |
| NF1_133-253-430 | 3 | E44 | c.6709C>T | |  | p.(Arg2237*) | Fahsold et al. 2000 | nonsense |
| NF1_52 | 1 | IVS44 | c.6756+1G>A | |  | p.(?) | Fahsold et al. 2000 | splicing |
| NF1_426 | 1 | IVS44 | c.6756+2T>C | |  | p.Phe2215fs | Pros et al. 2008 | splicing |
| NF1_458-182-**604** | 3 | E45 | c.6789_6792delTTAC | |  | p.(Tyr2264fs*4) | Robinson et al. 1995; Fahsold et al. 2000 | del |
| NF1_277-147 | 2 | E45 | c.6791insA | |  | p.(Tyr2264*) | Fahsold et al. 2000; Upaddhyaya et al. 1996; De Luca et al. 2004 | ins |
| NF1_246-350-**754** | 3 | E45 | c.6792C>A | | r.6792G>A | p.Tyr2264* | Robinson et al. 1995 | nonsense |
| **NF1_707** | 1 | E45 | c.6801A>G | | r.6757_6858del | p.Ala2253_Lys2286del | Valero et al. 2011 | splicing |
| NF1_257 | 1 | E47 | c.7096_7101delAACTTT |  | | p.(Asn2366_Phe2367del) | Abernathy et al. 1994 | del in frame |
| **NF1_695** | 1 | IVS47 | c.7126+3A>T | | r.7000_7126del | p.Glu2334fs*14 | Pros 2008 | splicing |
| NF1_90 | 1 | E48 | c.7190_7191delCT | |  | p.(Leu2398Glyfs*2) | Origone et al. 2002 | del |
| **NF1_740** | 1 | E50 | c.7395-2A>G | | r.7395_7552del | p.Tyr2466fs* | Brems et al. 2009 | splicing |
| NF1_331 | 1 | E52 | c.7720delA | |  | p.(Val2575Phefs*28) | Ars et al. 2003 | del |
| NF1_33-65-77 | 3 | E53 | c.7846C>T | |  | p.(Arg2616*) | De Luca et al. 2004; Upayaya et al. 1995 | nonsense |
| NF1_293 | 1 | IVS53 | c.7907+5G>A | |  | p.(?) | De Luca et al. 2004 | splicing |
| **NF1_527** | 1 | E54 | c.7993C>T | | r.7993C>U | p.Gln2665* | LOVD | nonsense |
|  |  |  |  | |  |  |  |  |

E= exon; IVS=intron; Del=deletion; Ins=insertion; Dup=duplication; **bold number=**patients screened through RNA approach
